# A comprehensive database of active and potentially-active continental faults in Chile at 1:25,000 scale

**DOI:** 10.1038/s41597-021-00802-4

**Published:** 2021-01-20

**Authors:** Valentina Maldonado, Martín Contreras, Daniel Melnick

**Affiliations:** 1 Instituto de Ciencias de la Tierra, TAQUACh, Universidad Austral de Chile, Valdivia, Chile; 2 Millennium Nucleus The Seismic Cycle Along Subduction Zones, Valdivia, Chile

**Keywords:** Natural hazards, Structural geology, Tectonics, Geomorphology

## Abstract

In seismically-active regions, mapping active and potentially-active faults is the first step to assess seismic hazards and site selection for paleoseismic studies that will estimate recurrence rates. Here, we present a comprehensive database of active and potentially-active continental faults in Chile based on existing studies and new mapping at 1:25,000 scale using geologic and geomorphic criteria and digital elevation models derived from TanDEM-X and LiDAR data. The database includes 958 fault strands grouped into 17 fault systems and classified based on activity (81 proved, 589 probable, 288 possible). The database is a contribution to the world compilation of active faults with applications among others in seismic hazard assessments, territorial planning, paleoseismology, geodynamics, landscape evolution processes, geothermal exploration, and in the study of feedbacks between continental deformation and the plate-boundary seismic cycle along subduction zones.

## Abstract (summary)

**Table:** 

Measurement(s)	active geological fault
Technology Type(s)	digital curation • remote sensing • satellite imaging of a planet
Factor Type(s)	fault with proved, probable and possible activity
Sample Characteristic - Environment	tectonically-active regions
Sample Characteristic - Location	Chile

Machine-accessible metadata file describing the reported data: 10.6084/m9.figshare.13268993

## Background & Summary

Despite the fact that geologists have been mapping active faults for more than a century^1,2^, the unexpected rupture of unknown or unmapped faults during recent large-magnitude earthquakes emphasizes our limited knowledge of the location and seismic potential of tectonically-active continental structures. During the past decade, at least five moment magnitude (M_w_) > 6 earthquakes have ruptures unknown continental faults. The 2010 Pichilemu, 2010 Darfield, 2011 Christchurch, 2016 Kaikoura, and 2019 Ridgecrest events involved ruptures on either completely concealed faults or unmapped fault strands^3–7^. These earthquakes highlight the need to identify and map active faults at detail scales as a first step in the assessment of seismic hazards associated with continental faults. In addition to environmental and seismic hazards, land-use and territorial planning, and civil protection emergency management, mapping active continental faults and classifying them into fault systems is important for different disciplines in earth sciences. These include: the evaluation of coastline migration and erosion; the exploration and exploitation of groundwater, geothermal, and other energy resources; the study of landscape evolution and the underlying erosive and tectonic mechanisms; and the search for feedback mechanisms between the seismic cycle of plate-boundary faults and continental deformation along subduction zones.

Active fault databases have been used for a broad range of applications in earth sciences. The primary application has been the assessment of seismic hazards^8^, which involves interpreting surface fault traces in terms of 3D seismic sources at depth^9–11^. Further applications have been, for example, assessing anthropogenic factors in triggered and induced seismicity^12^ such as stress changes to the crust caused by hydroelectric reservoirs, underground gas storage, groundwater pumping, or fracking^13,14^. Many geothermal fields occur in tectonically-active regions and maps of active faults have been used both for exploration and selection of drilling sites^15,16^ as well as for reservoir modelling during exploitation^17,18^. Active fault databases have been also used in studies of volcanotectonic interactions and structural control on volcanism in rifts^19^ and arcs^20^, in the interpretation of present-day stress indicators^21,22^ as well as to infer sources of pre-instrumental earthquakes^23^ and the response of groundwater to near and farfield earthquakes^24^. Analyses of the rupture mechanism, propagation and kinematics of many recent earthquakes and earthquake sequences have relied on databases of active faults derived from geomorphic and geologic data to interpret subsurface observations and develop conceptual models^3,6,25,26^.

Since the advent of modern instrumental seismology, earthquakes in Chile have accounted for >20% of the seismic moment release on Earth. The earthquakes accounted for in this estimate occurred along the megathrust fault that limits the Nazca and South American plates. However, the South American continental plate, as most upper-plates along subduction zones, includes numerous active faults, some associated with M_w_ > 6 earthquakes. Interestingly, the historical record of such *continental* earthquakes in Chile is relatively small, including only seven instrumentally-recorded events of M_w_ between 6 and 7 (Refs. ^4,27–29^). Out of these, only two (2001 Aroma and 2007 Aysén) occurred on mapped faults; another two were directly associated with larger earthquakes on the underlying plate-boundary megathrust (2010 Pichilemu and 2014 Pisagua) but occurred on unmapped faults. Only a few paleoseismic trenching studies have been carried out along four Chilean continental faults finding robust evidence for Mw > 6 paleoearthquakes^30,31^. It is however expected that the activity of further faults will be verified during forthcoming paleoseismic mapping and trenching endeavours. Recurrence periods of continental earthquakes estimated from paleoseismic and seismological studies are in the range of thousands of years (an order of magnitude larger than recurrence periods of megathrust earthquakes); nevertheless, considering the incipient knowledge of Chilean continental faults and their widespread spatial distribution, the seismic hazards posed by such structures should not be underestimated.

Research initiatives on active faults in Chile have so far only focused on specific faults or fault systems, and no unified and official database of active and potentially-active faults at national scale has been yet published. A regional assessment of neotectonic structures was first presented in the year 2000 including the first map of active faults and folds in Chile at 1:4,000,000 scale, as part of the World Map of Active Faults^32^; maps of this database were included in review papers addressing Quaternary deformation processes in South America^33,34^ and the neotectonics of Chile^35^. Subsequently, the South American Risk Project^36^, promoted by the Global Earthquake Model (GEM) project, incorporated those faults into a global database^37^. Recently, Santibañez *et al*.^38^ produced the first map of faults in Chile^38^ by compiling published studies including the 1:1,000,000 scale map of the Chilean Geological Survey^39^, and discussing the relation between regional tectonics, the recent instrumental crustal earthquakes, and mayor long-lived fault systems (active at >10^7^ yr timescales).

**Fig. 1:**
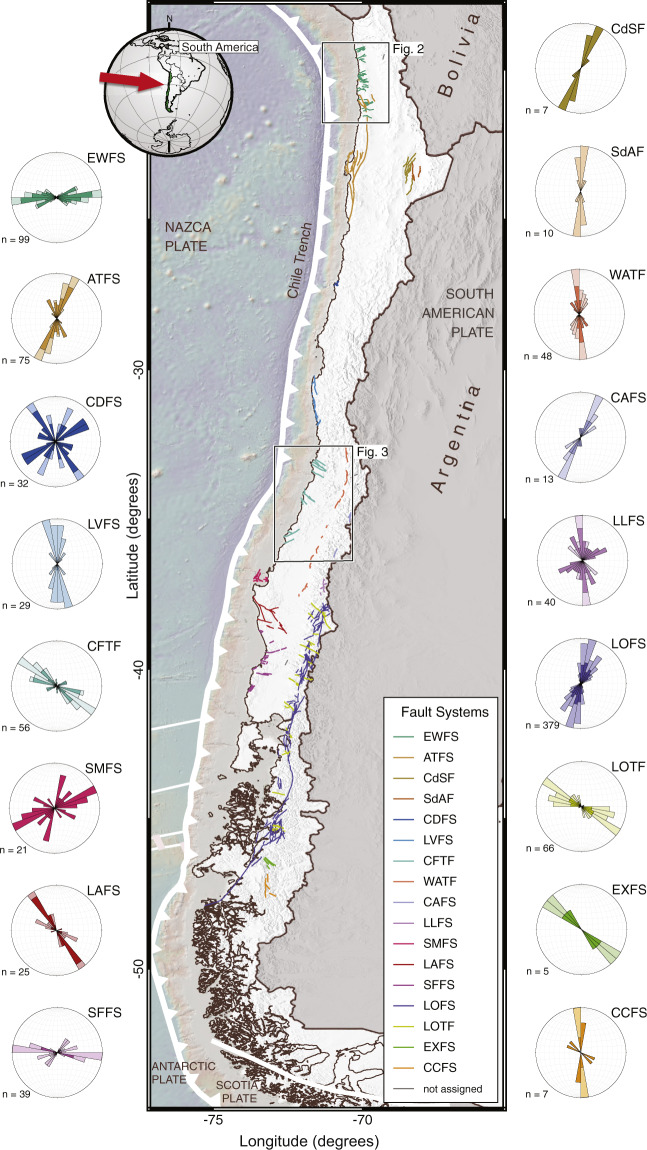
The CHAF database. Map of active and potentially-active faults colour-coded by fault system and shaded-relief topography from the SRTM30_plus dataset (http://topex.ucsd.edu/WWW_html/srtm30_plus.html). See Table 1 for Fault System names and basic statistics. Rose diagrams showing strike distributions for main fault systems (n = number of fault traces). Map made using QGIS 3.10 (www.qgis.org).

**Fig. 2:**
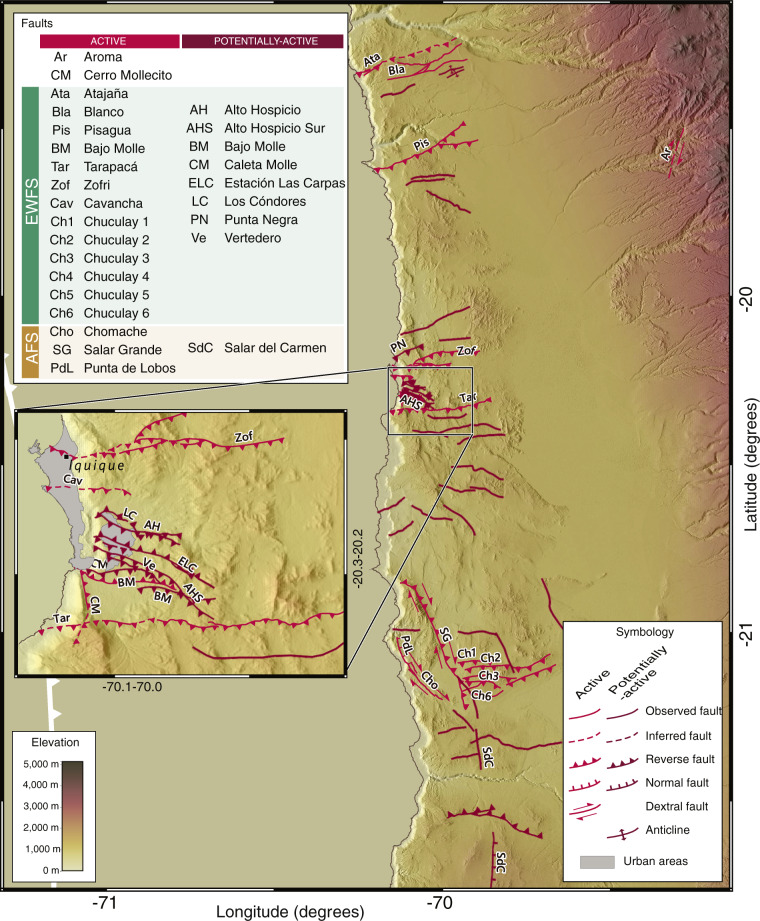
Example of the CHAF database in northern Chile. Shaded-relief topography from SRTM-1 dataset (https://dds.cr.usgs.gov/srtm/version2_1/SRTM1/). Inset shows detail of reverse faults in the vicinity of the Iquique urban area. Note persistent E-W strike of these faults, grouped into the EWFS (Table 1). Map made using QGIS 3.10 (www.qgis.org).

**Fig. 3:**
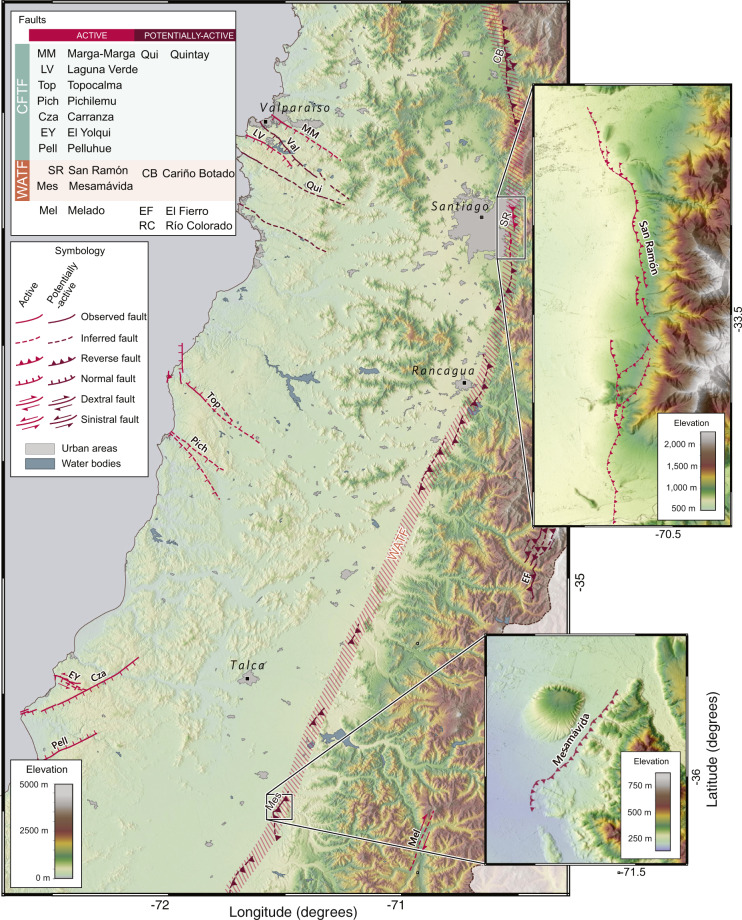
Example of the CHAF database in Metropolitan and central Chile. Shaded-relief topography from SRTM-1 dataset (https://dds.cr.usgs.gov/srtm/version2_1/SRTM1/). Upper inset shows thrust faults from the San Ramón Fault, part of the Western Andean Thrust Front fault system (Table 1), adjacent to the urban region of Santiago, Chile’s capital. Lower inset shows thrust faults of the Mesamávida Fault, a newly-identified structure from the same fault system. Map made using QGIS 3.10 (www.qgis.org).

Here, we present the CHilean Database of Active Faults (CHAF), a unified database of continental faults in Chile, within the South American continental plate (Figs. 1–3), which includes all the previous studies as well as newly-identified faults, using a common mapping scale and unified geomorphic criteria. We present basic statistics of fault and fault system geometrical characteristics, and a first-order estimate of maximum earthquake magnitudes using empirical relations. Our database is a contribution to the world compilation of active faults, with implications in various aspects of earth science research including geodynamics, volcanotectonics, paleoseismology, seismotectonics, studies of future earthquakes, exploration and exploitation of geothermal resources, structural control on landslides and volcanism, landscape evolution models, and seismic hazard assessments.

## Methods

### Fault mapping

Remote sensing data including aerial photographs, satellite images, and more recently Digital Elevation Models (DEM) have allowed the identification of geomorphic features as well as the application of quantitative morphological analyses to map topographic attributes with applications on active tectonic and structural geology studies^40–44^. We applied classical techniques in tectonic geomorphology summarized in seminal textbooks^45–47^ for mapping newly-identified faults and remapping structures from previous studies at a uniform 1:25,000 scale. We rely on our past experience in mapping active faults in different tectonic environments using field observations and remote sensing data^16,48–56^, in addition to the criteria used in previous active fault databases^57–61^. We paid special attention to interpreting fault trace continuity using a uniform mapping scale based on the surface expression of faults, not the inferred seismogenic expression at depth. The latter needs to be interpreted on the base of particular assumptions, and is therefore beyond the scope of our database. Our database is based on direct surface evidences. For mapping, we used hillshade and slope maps created using QGIS v. 3.10 (www.qgis.org) from DEMs derived from TanDEM-X data (12 m resolution) available for almost the entire region and from airborne LiDAR data (1 m, 2.5 m, and 5 m resolution) available along stretches of the Coastal Cordillera and along specific fault systems. TanDEM-X DEMs were provided by the German Aerospace Center (DLR) under Science Proposals GEOL0845, GEOL1209, GEOL1628, and GEOL0707 via the DLR science portal (https://tandemx-science.dlr.de/). LiDAR data was provided by Digimapas Chile and Forestal Arauco under collaboration agreements. Both datasets may be obtained from the authors on a reasonable request (see Usage Notes).

### Data classification and analysis

The database described in this study contains a line vector and metadata associated with each fault. The fields included in the metadata are reported in detail in the Data Records section. Faults in the CHAF database are grouped into fault systems and classified in terms of their estimated activity.

#### Fault system classification

**Fig. 4:**
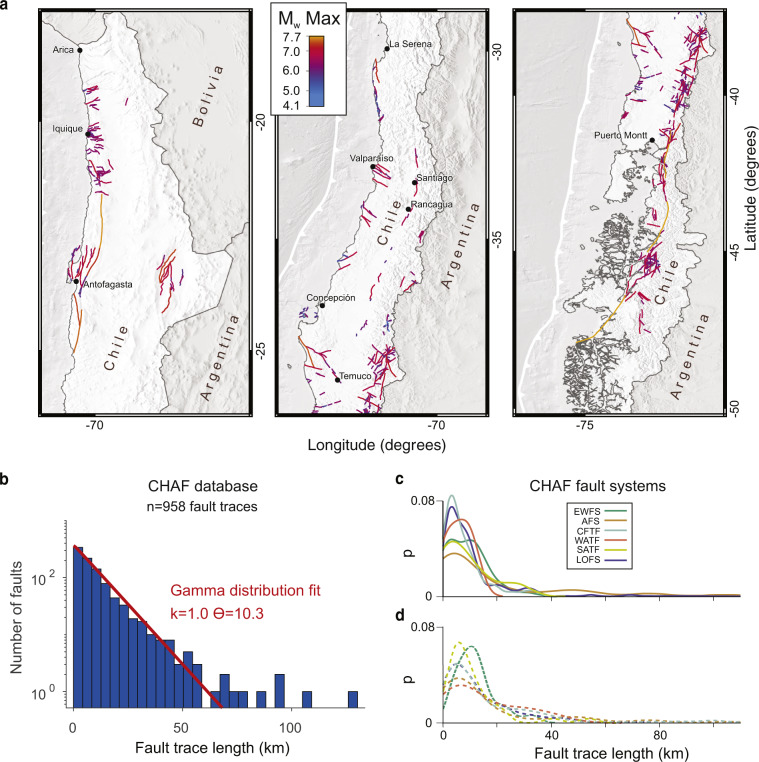
CHAF database statistics. (**a**) map of faults colour-coded by maximum earthquake magnitude expected for individual fault strands estimated using empirical relations^163^. Dots denote cities with more than 200,000 inhabitants. (**b**) distribution of fault lengths of the entire database. Note trade-off at ~60 km for gamma distribution fit. Faults with long traces belong to the LOFS and ATFS, both long-lived (>10^7^ years) strike-slip systems. (**c**) probability density functions of fault length for proven and probable faults grouped by fault system (p: probability density). (**d**) same as c for possible faults. Note similarity in fault length distributions for most fault systems.

We define a fault system as the population of faults distributed in a particular region that bear similarities in strike, kinematics, length distribution, and age. Fault system names have been considered on the base of previous studies, when existing (Table 1). Fault systems may or may not have a specific fault linkage geometry. For example, faults grouped into the CCTF and EWTS systems are kinematically but not geometrically linked, whereas faults grouped into the LOFS and LOTF are geometrically and kinematically linked (Fig. 1). In general, faults strands grouped in a fault system have similar strikes (Fig. 1) and fault length distributions (Fig. 4c, d). The defined fault systems may or may not be related to a certain bedrock unit and should not be considered as tectonic provinces, which encompass larger temporal and spatial scales.

**Table 1:** Fault systems of the CHAF Database.

Name	Code	No of faults	Mean strike	Number Proved faults	Number Probable faults	Number Possible faults	Fault System References*
Atacama	ATFS	75	72	29	34	12	^30,168^
Central Andean	CAFS	13	40	1	1	11	^96^
Cachet	CCFS	7	113	0	6	1	^56^
Caldera	CDFS	32	87	0	8	24	^62^
Central Coastal Forearc	CFTF	56	116	11	25	20	^52,54^, This study
Cordillera de la Sal	CdSF	9	23	0	0	9	^169^
East-West	EWFS	99	89	2	53	44	^66^
Exploradores	EXFS	5	134	0	0	5	^56^
Lanalhue	LAFS	25	134	0	20	5	^122^, This study
Lago Laja	LLFS	40	101	9	30	1	^48^
Liquine-Ofqui	LOFS	380	53	2	354	23	^49,153^
Liquine-Ofqui Transverse	LOTF	66	119	0	22	44	^142,153^
Los Vilos-Puerto Aldea	LVFS	29	105	0	2	27	This study
South-Central Coastal Forearc	SFFS	39	79	0	2	37	This study
Santa Maria	SMFS	21	66	1	20	0	^51,123,162^
Salar de Atacama	SdAF	10	74	0	10	0	^107^
Western Andean Thrust Front	WATF	48	97	25	1	22	^31,170^, This study
	not assigned	5	29	1	1	3	

*Key references of the Quaternary activity of the Fault System and definition of the fault system, for a complete reference list see the database and Supplementary File 1.

Fault traces have been assigned a *type* attribute based on four classes: (1) *blind*: faults that do not reach the surface as a break in the landscape, but may be associated with a fold or flexure; (2) *covered*: faults that are covered by undeformed young deposits; (3) *inferred*: faults whose surface expression is not clear and only estimated; and (4) *observed*: faults that have a clear surface expression at the 1:25,000 mapping scale.

Fault activity is classified as following. Active faults and folds are grouped in two categories: (1) *Faults with proved activity (Proved faults)*, those associated with an historical earthquake or with robust published evidence of slip (either seismic or aseismic) during the Holocene; or (2) *Faults with probable activity (Probable faults)*, those that exhibit direct geologic or geomorphic evidence of surface ruptures or deformation that allow to posit activity in the past 125,000 years. The age limit for *Probably active faults* is defined by the last interglacial period (Marine Isotope Stage 5e), when a distinct marine terrace was formed along most of the Pacific coastline^52,62^, which constitutes a suitable temporal geomorphic marker to observe fault offsets and classify fault activity. *Fault with possible activity (Possible faults)* integrate a third category, when geologic or geomorphic evidences of surface ruptures or deformation affecting the landscape allow to posit activity during the Quaternary period. For this latter case, deformed geomorphic markers include the fluvial network, pediment and alluvial surfaces, and glacial features.

## Data Records

The dataset presented here is stored in the Pangaea repository^63^, in ESRI Shapefile, Google Earth kmz, and spreadsheet xlsx formats. The dataset contains 958 records organized in 36 fields. Each record describes a single fault strand, which is part of a fault and grouped into a fault system. The 36 fields of the database may be grouped into: Identification (fields 1 to 8); Geometry (fields 9 to 19); Activity (fields 20 to 28); Historical seismicity (fields 29 to 32); Notes and references (33 to 36). The CHAF database includes 118 references^4,27,29,31,39,48,56,64–162^.

### Identification

1. **Fault trace id** (short name: F_id): Unique fault trace six-digit identifier, coded as “100101” (1/001/01: sector/fault/trace).
2. **Fault system** (short name: F_system): Mayor group of faults at regional scale.
3. **Fault name** (short name: F_name): Name of the main fault
4. **Trace name** (short name: FT_name): Name of the fault trace or segment
5. **Mapping data** (short name: map_data): Data used for fault mapping (TDX, LiDAR, Seismic reflection, microseismicity).
6. **Latitude** (short name: Lat): Latitude coordinate of the trace centre.
7. **Longitude** (short name: Lon): Longitude coordinate of the trace centre.
8. **Fault type** (short name: type): Interpreted fault (observed, inferred, covered, blind).

### Geometry

9. **Geometry class** (short name: geom): structure geometry (simple fault, segmented fault, fold).
10. **Strike** (short name: strike): Strike of the fault trace.
11. **Dip** (short name: dip): Dip of the fault trace.
12. **Dip direction** (short name: dipdir): Dip direction of the fault trace.
13. **Rake** (short name: rake): Rake of the main fault.
14. **Sense of movement** (short name: sense): Sense of movement of the main fault.
15. **Fault trace length** (short name: length_km): Along-strike length of the fault trace.
16. **Minimum depth** (short name: min_z_km): Minimum depth of the fault trace.
17. **Maximum depth** (short name: max_z_km): Maximum depth of the fault trace.
18. **Width** (short name: width_km): Down-dip width depth of the fault trace.
19. **Area** (short name: area_km^2^): Fault area in km^2^.

### Ac**t**ivity

20. **Age of activity** (short name: age): This field includes four classes: Historic; Holocene; Late Quaternary (<125 ka); Quaternary.
21. **Activity class** (short name: activity): This field includes three classes: proved (based on paleoseismic trenching or historical activity); probable (based on geomorphic criteria and/or unequivocally aligned crustal microseismicity); possible (geomorphic criteria).
22. **Recent seismic activity** (short name: recent_act): Most-recent evidences of faulting and displacement.
23. **Faulting onset** (short name: onset): Estimated age for the onset of faulting.
24. **Throw rate** (short name: throw_rate): Vertical slip rate in mm/yr.
25. **Horizontal slip rate** (short name: h_slip_rate): Horizontal slip rate in mm/yr.
26. **Slip rate** (short name: slip_rate): Fault slip rate in mm/yr.
27. **Aseismic slip evidence** (short name: aseismic_slip): Evidences for aseismic slip from paleoseismic or geodetic data.
28. **Paleoseismic evidence** (short name: paleo_ev): Evidences of past earthquakes from paleoseismic data.

### Historical se**i**smicity

29. **Associated seismicity** (short name: ass_seim): Related microseismicity or historical earthquakes.
30. **Maximum registered magnitude** (short name: Mmax_r): Maximum magnitude of an instrumentally-recorded earthquake in moment tensor magnitude scale.
31. **Maximum estimated magnitude** (short name: Mmax_e): Maximum magnitude of an estimated earthquake in moment tensor magnitude scale.
32. **Recurrence interval** (short name: rec_int): Earthquake recurrence interval of the maximum recorded or estimated magnitude.

### Not**e**s and references

33. **Notes** (short name: notes): Notes and comments.
34. **Trace source** (short name: source): Source of the fault trace (ref: digitized from published study or studies; Unif: re-mapped based on a published study; Map: based on new geomorphic interpretations made in this study).
35. **Source mapping scale** (short name: map_scale): Scale of the source map, in case of a previously-mapped fault.
36. **References** (short name: refs): Published studies used as reference for mapping and/or metadata. A complete list of all the references used in the database is provided in the Supplementary File 1.

### Statistical data analysis

We grouped fault traces of the CHAF database into 17 Fault Systems (Table 1). The comprehensive mapping scale of the database allows estimating first-order statistics of fault traces and fault systems that may be relevant and of interest to various different disciplines in Earth sciences. Empirical relations estimated from the surface rupture length and magnitude of historical earthquakes^163^ provide a first-order assessment of seismic hazard implications from the CHAF database (Fig. 4a). The distribution of fault length of the entire database (Fig. 4b) suggests that faults are self-similar until a length of ~60 km. Longer traces might require higher-resolution topography and/or mapping at a detailed scale for subdivision, or might reflect mature faults that have accumulated larger magnitudes of deformation resulting in higher geometrical connectivity.

## Technical Validation

The CHAF database is difficult to validated by any designed experiment. The metadata of our database follows criteria established and validated by governmental institutions of different countries (i.e., USGS^164^, GNS^165,166^, AIST^167^, GEM^57,61^, and CCAF^60^). The most important validation procedure will be the occurrence of a forthcoming earthquake on a mapped and properly-classified fault. However, another validation procedure is the comparison with independently-made maps published in previous studies. All these references used in the compilation of the active fault database are included as “Codes” in the digital files, allowing to check the original publication and compare the fault traces. The complete list of references in the database is provided also separately in the Supplementary File 1. The original DEM data may be provided from the authors based on reasonable request, for any project that seeks an independent validation procedure.

We validate the grouping of individual mapped fault traces into fault systems by analysing the variation in fault strike (Fig. 1) and fault length (Fig. 4b–d). All the individual fault systems have similar length distributions, both for probable and possible faults (Fig. 4b).

The present first version of the CHAF database is intended to be the start of a long-term community-based project. To achieve this goal, we created the website www.fallasactivas.cl that includes a map server to visualize the fault traces, fault systems, and associated metadata. Satellite imagery and hillshade maps created from the DEMs used for mapping have been also included. The website contains a blog aimed at obtaining feedback from the community and to allow for the submission of relevant new data on mapped faults or newly-identified unmapped faults, to update the database.

## Usage Notes

The LiDAR data used for mapping previously- and newly-identified faults was in part acquired from the company Digimapas Chile and in part donated by Forestal Arauco to the CYCLO project under a confidentiality agreement. The data may be obtained from the corresponding author based on a reasonable request and a Memorandum of Understanding (MoU); a draft MoU may be found in the Supplementary Materials. TanDEM-X DEMs may be obtained from the corresponding author based on a reasonable request and from the German Aerospace Center (DLR).

## Supplementary information

Supplementary File 1
