# Supplementary material for: A comprehensive database of active and potentially-active continental faults in Chile at 1:25,000 scale
Source: Sci Data. 2021 Jan 20;8:20. doi: 10.1038/s41597-021-00802-4 (PMC7817672; doi:10.1038/s41597-021-00802-4)
Supplement: Supplementary file 1 [file 41597_2021_802_MOESM1_ESM.docx]

**LiDAR Data-Sharing and Usage Agreement**

**Universidad Austral de Chile and Millennium Nucleus CYCLO**

This agreement establishes the terms and conditions under which the Universidad Austral de Chile (UACh) via the Director of the Millennium Nucleus CYCLO can share LiDAR (Light Detection and Ranging) data in the form of raw Digital Terrain Model (DTM) grids to the other party. UACh and CYCLO will act as data provider and the other institution as data recipient.

1. The confidentiality of data will be protected as follows:
2. The data recipient will not be released to individuals but to a recipient institution.
3. The data will remain property of the private company that donated the data to UACh and CYCLO.
4. The data recipient will not release data to a third party without prior approval from the data provider.
5. The data will be provided exclusively for research activities, and not for economic or business purposes.
6. Researchers from the data recipient institution should detail the purpose of the activities involving the data to the representative of the provider institution.
7. If applicable, the data transfer may lead to collaborative research activities involving training of students and joint publications.
8. The data will be transferred to the recipient prior authorization from the data owner.
9. The data recipient will not share, publish, or otherwise release any findings or conclusions derived from analysis of data obtained from the data provider without prior approval from the data provider.
10. Data transferred pursuant to the terms of this Agreement shall be utilized solely for the purposes set forth in the “Partnership Agreement”.
11. Any third party granted access to data, as permitted under condition #2, above, shall be subject to the terms and conditions of this agreement. Acceptance of these terms must be provided in writing by the third party before data will be released.

IN WITNESS WHEREOF, both the Universidad Austral de Chile and Millennium Nucleus CYCLO, through its duly authorized representative, and the __________________________ (data recipient Institution), through its duly authorized representative, have hereunto executed this Data Sharing Agreement as of the last date below written.

Dr. Daniel Melnick

**Professor, Universidad Austral de Chile**

**Director, Millennium Nucleus CYCLO**

Date:

Legal representative of “Data recipient” institution.

Date:
